# Common Themes and Future Challenges in Understanding Gene Regulatory Network Evolution

**DOI:** 10.3390/cells11030510

**Published:** 2022-02-01

**Authors:** Isabella Schember, Marc S. Halfon

**Affiliations:** 1 Department of Biochemistry, University at Buffalo-State University of New York, Buffalo, NY 14203, USA; ilschemb@buffalo.edu; 2 Department of Biomedical Informatics, University at Buffalo-State University of New York, Buffalo, NY 14203, USA; 3 Department of Biological Sciences, University at Buffalo-State University of New York, Buffalo, NY 14260, USA; 4 NY State Center of Excellence in Bioinformatics & Life Sciences, Buffalo, NY 14203, USA; 5 Department of Molecular and Cellular Biology and Program in Cancer Genetics, Roswell Park Comprehensive Cancer Center, Buffalo, NY 14263, USA

**Keywords:** gene regulatory networks, evolution, *cis*-regulatory modules, enhancers, *Drosophila*, *Heliconius*, pigmentation pattern, modularity, co-option

## Abstract

A major driving force behind the evolution of species-specific traits and novel structures is alterations in gene regulatory networks (GRNs). Comprehending evolution therefore requires an understanding of the nature of changes in GRN structure and the responsible mechanisms. Here, we review two insect pigmentation GRNs in order to examine common themes in GRN evolution and to reveal some of the challenges associated with investigating changes in GRNs across different evolutionary distances at the molecular level. The pigmentation GRN in *Drosophila melanogaster* and other drosophilids is a well-defined network for which studies from closely related species illuminate the different ways co-option of regulators can occur. The pigmentation GRN for butterflies of the *Heliconius* species group is less fully detailed but it is emerging as a useful model for exploring important questions about redundancy and modularity in *cis*-regulatory systems. Both GRNs serve to highlight the ways in which redeployment of *trans*-acting factors can lead to GRN rewiring and network co-option. To gain insight into GRN evolution, we discuss the importance of defining GRN architecture at multiple levels both within and between species and of utilizing a range of complementary approaches.

## 1. Gene Regulatory Networks and their Architecture

**Figure 1:**
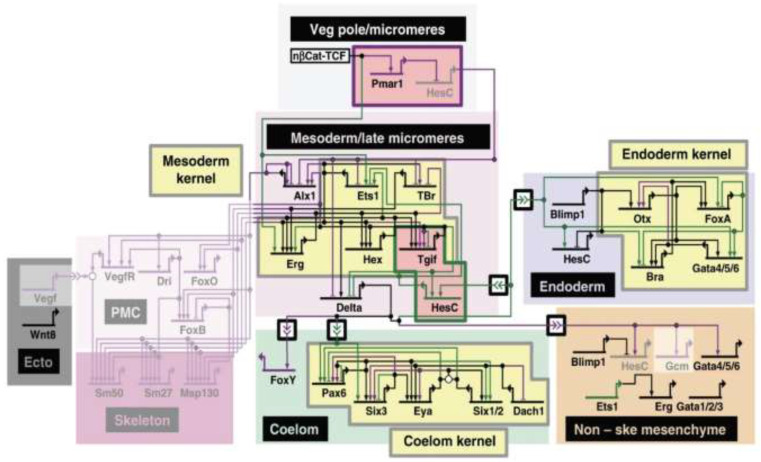
Gene regulatory network showing conserved kernels and both shared and species-specific subcircuits. A schematic of the GRN for endomesoderm specification for both sea urchin and sea star is depicted (see ref. [7] for details). Genes are shown in the different regions (colored boxes) where they are expressed during development. Activating inputs are represented by arrows and repressive inputs by bars. Intercellular signaling is shown using double arrowheads. Purple genes and linkages are unique to sea urchin, while green is specific to sea stars, and black are those in common. The “kernels” (yellow) and distinct subcircuits (pink) are highlighted. The greyed-out backgrounds indicate network circuits absent in sea stars. Image credit: © Cary et al. [7], used with permission under CC-BY 4.0 license.

Gene regulatory networks (GRNs) provide a potent framework for conceptualizing the interactions of the genes, regulatory proteins, and signaling pathways that comprise the coordinated gene expression programs at the root of both embryonic and postembryonic development [1,2,3,4]. GRNs are structured as interconnected modular components with a hierarchical structure [1,2,4,5]. The nodes of a GRN consist of genes and their *cis*-regulatory modules (CRMs), which control the spatio-temporal patterns of gene expression, while *trans*-acting transcription factors (TFs) and signaling pathways serve as the network “edges” (Figure 1) [1,2,4,6].

GRN modularity increases in tandem with developmental complexity: as cell lineages grow in number but restrict in potential, the GRNs correspondingly divide into submodules (often referred to as “sub-circuits”) with distinct regulatory functions (Figure 1). The subcircuits themselves are tightly connected networks of regulatory linkages that interact across tiers of the GRN (Figure 1). The hierarchy of GRN constraint is inversely related to developmental potential, from largely inflexible “kernels” specifying essential developmental fields, through conserved “plug-in” modules of signal transduction pathways that are used as parts of multiple different GRNs, down to the highly labile “differentiation gene batteries” responsible for cell type-specific processes (Figure 1) [1,2,4,8,9,10,11]. Changes in kernels are predicted to have a large and pleiotropic effect with significant evolutionary consequences, driving phenotypic diversity and eventually speciation; this accounts for their relative evolutionary stability. Changes in more terminal subcircuits, on the other hand, may have little or no phenotypic impact, leaving them free to diversify extensively [2,5,8,9,11].

Identifying the primary mechanisms responsible for evolutionary changes in GRNs is a topic of intense current interest. A consistent challenge lies in the fact that while studying GRN evolution in a detailed way requires defined GRNs with well-characterized CRMs and TFs in two or more related species [6,11], there are few sufficiently detailed examples. Ideally, each species would have distinct morphological and other phenotypic differences. Understanding the relationships between these structures and traits in terms of their homology is an additional important step in defining the mechanisms responsible for a given GRN’s evolutionary changes [8], as elegantly shown in the extensive comparison of the GRNs responsible for sea urchin and sea star endomesoderm specification [7,9]. While there are entire subcircuits that are specific to sea urchins, there are also common subcircuits and highly conserved kernels (Figure 1). Understanding homology is particularly important when looking at co-option, where subcircuits are re-deployed in different developmental contexts to adapt to a new purpose [6,8]. The ease with which a subcircuit can be co-opted and the consequences of such an event are dependent on where the subcircuit lies in the GRN hierarchy [8].

In the paragraphs that follow, we use two insect pigmentation GRNs to illustrate common themes in GRN evolution and to highlight some of the challenges faced when attempting to understand GRN evolution at a molecular level. Studies of the well-defined *Drosophila* abdominal and wing pigmentation subcircuits reveal insights into the varied ways in which regulators can be co-opted, but they suggest that certain mechanisms might be more prevalent than others. The description of the wing pigmentation GRN in *Heliconius* butterflies is currently at a more elementary state, but recent studies of this GRN illustrate features of evolution at a higher tier in the GRN structure and challenge our understanding of CRM modularity. We discuss how studies of GRN evolution can be enhanced by having a broad toolbox of both traditional and contemporary methods and a perspective from multiple GRN levels for GRNs of similar function from a range of closely and more distantly related species.

## 2. Evolution of the Drosophila Pigmentation GRN

**Figure 2:**
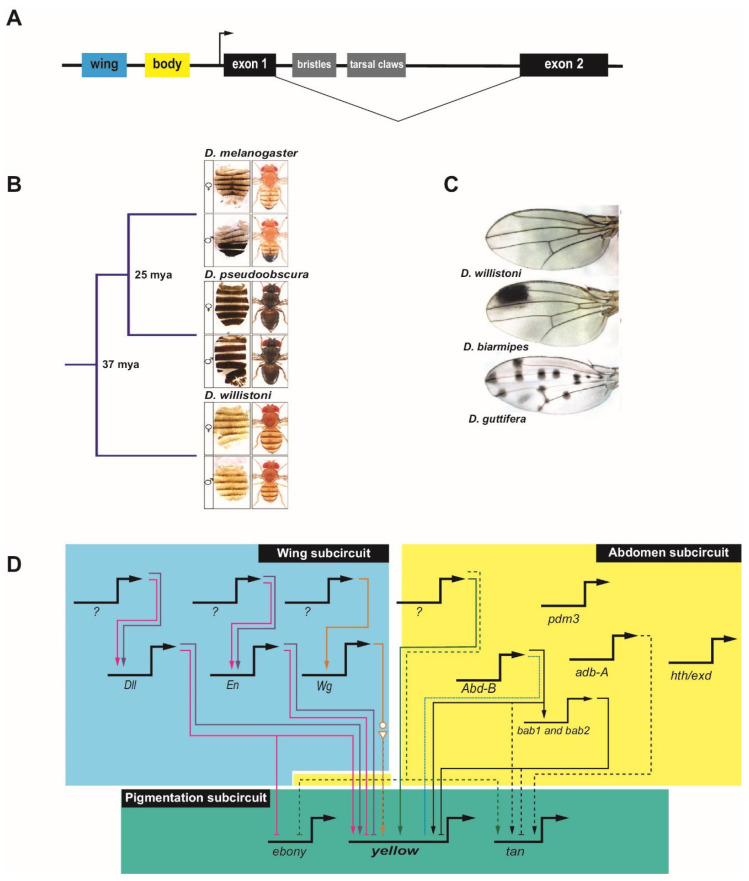
The *Drosophila* pigmentation gene regulatory network subcircuit. (**A**) Schematic of the *yellow* locus, with the positions of the ‘body element’ and ‘wing enhancer” highlighted. The bristle and tarsal claw CRMs are shown in grey. Note that additional regulatory sequences, not pictured, can be found throughout the locus, including in the large intron (drawing not to scale). (**B**) The differences in abdominal pigmentation and the phylogenetic relationship between three species, (top to bottom) *D. melanogaster*, *D. pseudoobscura*, and *D. willistoni*. From left to right: dissected dorsal abdomen and dorsal view of adult fly for males and females. (**C**) Wings from *D. willistoni*, *D. biarmipes*, and *D. guttifera*, showing differences in pigmentation pattern. (**D**) Partial schematic of the *Drosophila* pigmentation GRN, with emphasis on *yellow*; see text for details. The regulation of *yellow*, *ebony*, and *tan* by upstream factors is either direct (solid lines) or indirect (dashed lines) and can be in the form of activating (arrow) or repressive (bar) inputs. Species-specific loss of binding is shown using a dotted line. Unknown upstream factors are denoted by question marks. The wing (blue box) and abdomen (yellow box) subcircuits are shown separately, even though they share components, to better illustrate the regulatory differences in these tissues. The abdomen subcircuit focuses on regulation in the A5 and A6 segments. Multiple species have been incorporated into one network; however, the individual CRMs involved are not depicted. Although additional linkages can be inferred, for simplicity only those discussed in the text are included. Linkages unique to *D. melanogaster* are colored black, those specific to *D. prostipennis* are colored green, those specific to *D. kikkawai* are colored blue, those specific to *D. biampries* are colored pink, those specific to *D. elegans* are colored purple, and those specific to *D. guttifera* are colored orange. Image credits: panel B, © Kalay, et al. [19] used with permission under CC-BY 4.0 license; panel C, reprinted from Rebeiz and Williams [13], © Elsevier, used with permission.

GRN evolution has been well studied in the genus *Drosophila*, and much of our understanding of modularity in regulatory evolution comes from studies in *Drosophila* species. The diverse pigmentation patterns of the wings and bodies of *Drosophila melanogaster* and its close relatives provide a favorable model for studying the evolution of CRM sequences [12,13,14]. The underlying genetics of pigmentation is well established. Pigmentation in both the abdominal cuticle and the wing is associated with genes that regulate melanin production (reviewed by ref. [13]). The gene *yellow* is required for black melanin formation [15,16], while *ebony* encodes an enzyme that promotes the yellow (non-melanic) cuticle [16,17]. Both *yellow* and *ebony* are required for pigmentation in the wing (reviewed by ref. [13]), while in the abdomen an additional gene, *tan*, is expressed in a pattern coincident with *yellow* in males to promote the darker male cuticle coloration [18]. Upstream regulators responsible for the patterns of *yellow*, *tan*, and *ebony* have been identified and the main CRMs for these genes are known, leading to identified subcircuits for pigmentation [13]. Particularly well-defined are the inputs to the gene *yellow* in the GRN subcircuits controlling wing and abdomen pigmentation (Figure 2). 

Expression of *yellow* in the wing, head, thorax, abdomen, and bristles is under the control of four major tissue-specific CRMs in the upstream 5′ regulatory region and single intron of the gene (Figure 2A) [15,20,21]. Two main separable CRMs in the gene’s upstream regulatory region mediate abdomen and wing expression—the ‘body element’ and the ‘wing enhancer’, respectively—and diverged pigmentation has been directly correlated with evolutionary changes in these CRMs (Figure 2A–C) [12,15,16,21,22,23]. 

Changes at the *cis* level in the *yellow* locus in the *Drosophila* pigmentation GRN lead to changes in black melanic pigmentation by modulating levels of *yellow*. For instance, *cis* changes within the ‘body element’ CRM [16] can lead to the loss of *yellow* expression. In the *melanogaster* species group, male-specific abdominal pigmentation is partially controlled through the action of the Hox protein Abd-B, which binds to the ‘body element’ CRM in most species of this group to activate *yellow* transcription via a conserved binding site [24]. However, in *Drosophila kikkawai*, which lacks pigmentation, changes in this site eliminate Abd-B binding, explaining the loss of *yellow* expression (Figure 2D). Given the established importance of Abd-B for regulation of the ‘body element’ CRM, the loss of the Abd-B binding site was likely the causative event leading to loss of *yellow*, although subsequent functional changes are also apparent; restoration of the Abd-B binding site alone is not sufficient to restore activity of the *D kikkawai* CRM when used in a reporter gene experiment in transgenic *D. melanogaster* [24]. Similarly, epistasis resulting from inactivation of Abd-B-responsive CRMs for *yellow* (as well as *tan* and *ebony*) in *Drosophila santomea* insulates *D. santomea* abdominal pigmentation from the effects of changes in Abd-B expression [25]. Interestingly, the ‘body element’ CRMs of several other species appear to have retained Abd-B binding capability and the ability to respond to Abd-B to drive male-specific abdominal expression when transposed into *D. melanogaster*, despite having lost the pigmentation trait itself. This suggests that in these species, response to Abd-B is being regulated at an earlier point in the GRN. One such point may be regulation of the repressor Bric-à-brac (Bab), which in *D. melanogaster* binds to the ‘body element’ CRM to repress *yellow* transcription in the female abdomen [26]. Abd-B is a direct activator of *bab* through binding at multiple sites in a *bab* CRM [27], suggesting one possible mechanism by which *yellow* expression could be repressed despite ability of the ‘body element’ CRM to bind Abd-B (Figure 2D). The Bab-dependent regulation of the pigmentation gene *tan* may not occur via direct binding to the known *tan* CRM, and it may instead reflect an indirect *trans*-regulatory effect [26]. Thus, multiple modes appear to be employed to reach a common evolutionary change in pigmentation. Direct targets of additional TFs and their positions in the abdomen subcircuit of the pigmentation GRN have not yet been established [28,29,30].

Additionally, instances of trait gain are a result of *cis* changes in *yellow* CRMs. Expansion of melanic pigmentation into more anterior abdominal segments in *Drosophila prostipennis* has been mapped to activating changes in a *cis*-regulatory sequence in the region of the combined wing and body CRMs [31]. Surprisingly, similar activating changes in *tan* expression in this same species and reciprocal loss of *ebony* expression appear due to *trans*, rather than *cis*, effects (Figure 2D). Therefore, seemingly coordinated regulatory evolution can result from disparate mechanisms, as discussed above [31]. Kalay et al. [32] examined instances of trait loss and gain through changes in *yellow* expression leading to the overall dark pigmentation on the abdomen and thorax of *Drosophila pseudoobscura* (Figure 2B) and the more limited thoracic pigmentation of abdominal segments in *D. melanogaster* and *Drosophila willistoni* (Figure 2B). They found that extensive redundancy exists among the *cis*-regulatory sequences that drive *yellow* expression, with many sequences in addition to the previously well-characterized wing and ‘body element’ CRMs able to drive similar wing and abdominal expression. Similarly, Xin, et al. [33] identified important regulatory activity in sequences adjacent to the originally defined ‘wing spot’ enhancer. This finding highlights one of the challenges of tracing GRN evolution: when there are multiple CRMs, there are multiple places where activating mutations can exert an effect and multiple sequences that must be examined to pinpoint the relevant changes. Interestingly, Kalay et al. [32] also observed what they termed “cryptic” CRM activity in which enhancer fragments in transgenic reporter assays drove expression patterns that longer versions of the same enhancer did not. Frequently, the cryptic patterns were consistent with patterns of *yellow* expression seen in other *Drosophila* species, suggesting these sequences possess the potential to drive such patterns but are repressed by neighboring sequences in the genome. This is consistent with recent findings suggesting that gain of repressive sequences may precede the evolution of activating sequences [34].

Similar to what has been observed for abdominal pigmentation, male-specific wing pigmentation patterns have also been gained and lost multiple times in the *Drosophila* clade. Again, a number of changes have been mapped to *cis*-level variation in *yellow*. Loss of *yellow* expression in *Drosophila gunungcola* wings is the result of two to seven point mutations in the ‘wing’ CRM, affecting as yet unidentified regulators (not pictured) [22]. Male-specific pigmented spots in *Drosophila biarmipes* (Figure 2C) and *Drosophila elegans* wings are due to the evolution of specific binding sites near or within this CRM, including acquisition of sites for the activator Distal-less (Dll) and the repressor Engrailed (En) (Figure 2D) [14,22,35]. Dll is also responsible for reciprocal repression of *ebony*, although it is not clear whether this is a direct or indirect effect at the level of *ebony cis*-regulation [14]. Moreover, the expression pattern of *Dll* itself has been modified in wing-spot bearing species. Analysis of multiple species suggests that incorporation of *cis*-responsiveness to Dll emerged first, followed by elaboration of the new pigmentation patterns allowed through diversification of the *Dll* expression pattern. 

In *Drosophila guttifera,* a newly-evolved “vein spot” CRM promotes expression of *yellow* in what will become 16 melanic spots in the adult wing (Figure 2C). This CRM is indirectly regulated through the activity of the Wingless (Wg) (Wnt) signaling pathway; *wg* itself has acquired new regions of expression through modification of two wing-specific CRMs (Figure 2D) [23,36]. This situation is strikingly similar to that described above for Dll-mediated regulation of the *D. biarmipes* wing spot: in both cases, *cis*-level incorporation of the ability to respond to a new regulator has been followed by expansion of the expression of the regulator into new domains. Notably, Dll and Wg are both regulators that are essential morphogenetic patterning genes in the wing. Similarly, the elaborately black-and-white patterned wings in the related fly genus *Samoaia* appear to have independently co-opted both *Dll* and *en* into the pigmentation GRN [37]. Although the CRM-level details have not been established, the correlated changes in *en* expression and wing-spot location suggest that here, too, incorporation into the GRN preceded redeployment of the *trans*-acting developmental regulator. This suggests a common and an effective route for evolving morphological novelties by first modifying the *cis*-responsiveness of a GRN’s downstream effector genes, followed by redeployment of more upstream patterning genes to produce spatial diversification of the trait [14].

Although this GRN has been extensively studied, the studies have used a variety of *Drosophila* species, sometimes with different sets of features examined. This makes it challenging to understand if and where common mechanisms are responsible for the changes in pigmentation seen across *Drosophila* and to follow the trajectory of *cis*-regulatory changes across the clade. A comprehensive approach in which an identical set of genetic and sequence-level assays was applied to a carefully selected range of species would be a benefit in this regard, to allow a clearer view of the *cis*- and *trans*-changes responsible for evolution of *yellow* regulation and the pigmentation GRN. 

## 3. Evolution of the *Heliconius* Pigmentation GRN

**Figure 3:**
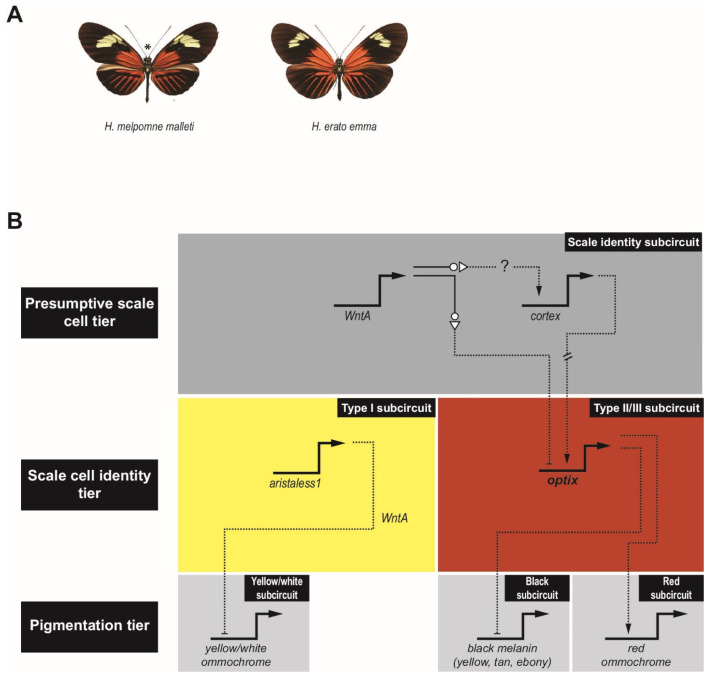
The *Heliconius* pigmentation gene regulatory network subcircuit. (**A**) Wings of *H. melpomene malleti* (left) and *H. erato emma* (right) reveal their color pattern mimicry. (**B**) Proposed *Heliconius* pigmentation GRN; see text for details. The three subcircuits (grey boxes) in the pigmentation tier (bottom) are the yellow/white subcircuit, the black subcircuit, and the red subcircuit. The scale identity tier (middle) has two main subcircuits that have been identified, the Type I subcircuit (yellow box) and Type II/III subcircuit (red box). The top tier of the GRN specifies the presumptive scale cells (upper grey box). Regulation by upstream factors is hypothesized based on existing genetic data; direct TF-CRM relationships remain to be established. Arrows indicate activation and bars, repression. Image credit: panel A, © Wallbank et al. [56], used with permission under CC-BY 4.0 license.

Significant insights into GRN evolution are also starting to emerge from studies of wing pigmentation in *Heliconius* butterflies. These butterflies have a melanin synthesis pathway similar to that of *Drosophila*, i.e., with *yellow, tan,* and *ebony* orthologs, as well as ommochrome pigment synthesis pathways responsible for red, orange, and yellow coloration [38]. *Heliconius* are examples of Müllerian mimicry, where local populations evolve to imitate the color patterning of local toxic butterflies and moths [39,40,41,42,43,44]. Two well-studied *Heliconius* species are *Heliconius erato* and *Heliconius melpomene,* which have converged on similar wing patterns (Figure 3A) [40,45]. In marked contrast to the situation described above for *Drosophila*, where *cis*-regulatory changes at the level of the pigmentation-synthesis enzymes appear to account for a significant portion of observed variation in pigmentation pattern, genetic studies in *Heliconius* suggest that responsibility for pigmentation shifts lies elsewhere [38]. Most changes have been mapped to just four major effect pigment patterning genes: *optix*, *cortex*, *aristaless1*, and *WntA* (Figure 3B) [40,46,47,48,49,50,51]. Responsible for establishing wing scale cell identity and competence to respond to TFs more directly involved in pigmentation (reviewed by ref. [51])), *cortex* and *WntA* act at a higher tier in the GRN (Figure 3B, top): *cortex* is necessary to specify “Type II/III” scales [52]; and *optix* is responsible for further differentiation into Type III scales and promotion of red ommochrome production, while repressing the melanin pathway [53] (Figure 3B, middle right). In the absence of *cortex,* cells take on the “Type I” fate in which *aristaless1* dictates white or yellow pigmentation via repression of yellow ommochrome production [54] (Figure 3B, middle left). The role of *WntA* is complex and varied [55] but modulation of *optix* expression is at least one downstream consequence. In some species, *WntA* appears to act like a “shutter,” an upstream repressor responsible for differential expression, ultimately controlling where color appears on the forewings [39]. The observed phenotypic variation in the butterfly wing therefore appears primarily due to differences in the expression of a small set of upstream regulators, only two of which—*optix* and *aristaless1*—are transcription factors, although additional *cis*-level changes in some of the downstream genes cannot be ruled out. As in *Drosophila*, where wing patterning genes such as *dll, en*, and *wg* were co-opted into the pigmentation GRN, *optix* and *WntA* also play major roles in patterning the morphology of the wing. The evidence in *Drosophila* suggests that co-option occurred first, followed by diversification of spatial patterning, as discussed above. It remains to be determined whether or not a similar scenario holds for *Heliconius*.

The diversity in *Heliconius* wing patterns is most clearly understood for red pigmented regions. For these, differences can largely be attributed to changes in the *optix* locus [39,40,49,56], which alter the expression of downstream genes and transcription factors that are involved in pigment patterning [40,57]. Recent work by Lewis et al. [39] investigated the *cis*-regulatory architecture at the *optix* locus by using CRISPR/Cas9 to disrupt specific *optix* CRMs. This approach resulted in three significant findings, none of which would have been obvious from a more traditional reporter-gene based CRM discovery approach: (1) the CRMs are pleiotropic, with the CRM mutations resulting in multiple phenotypic effects on both wing vein development and pigmentation; (2) many CRMs appear to act as both silencers and enhancers, that is, they may be capable of both repressing and activating *optix* expression in different positional contexts; and (3) surprisingly, the CRMs in the locus are interdependent, such that perturbation of any CRM could cause widespread loss of red color pattern.

The ability of *optix* CRMs to act as both silencers and enhancers is in keeping with a growing realization that CRMs can have multiple regulatory functions (reviewed by [58]). The observed silencer activity suggests that these sequences could play a role in integrating *WntA* inputs to achieve the “shuttering” effects observed by Lewis et al. [39] in which a broad potential domain of *optix* expression is modulated by region-specific silencing to suppress red pigmentation. It will be interesting to determine how this mechanism relates to the “cryptic” enhancer activity observed in the *D. melanogaster yellow* locus above [32]—do those sequences also represent dual-function “shuttering” silencers?—as well as to explore the evolutionary history of which became incorporated into the GRNs first, the activating or the silencing activities.

The finding that the *optix* CRMs are interdependent runs counter to the canonical view of CRMs as being strictly modular and it argues that multiple CRMs are necessary, and not merely acting redundantly, to establish regions of red pigmentation. This extensive CRM interdependence is also surprising given that population genetics analyses suggest that introgression of CRM sequences and CRM shuffling have played notable roles in generating adaptive variation across *Heliconius* species [56,59,60]. Resolving these discrepancies should be an important focus for future research.

Is the interdependency of *optix* CRMs unique to *Heliconius*, or is it something that has gone unnoticed in other GRNs? Most previous CRM-level studies have been performed using extensive reporter gene analysis, whereas Lewis et al. [39] deleted CRMs using CRISPR/Cas9. These two assays give different types of information and can lead to different interpretations [61]. Had Lewis et al. used reporter genes instead of knockouts, they may well have simply concluded that the *optix* locus contained multiple modular, partly redundant CRMs—a view more consistent with previous genetic studies—in a manner similar to that described for the *D. melanogaster yellow* locus, as discussed above [32]. However, this raises the question of whether had Kalay et al. [32] conversely undertaken a perturbation analysis of the *yellow* locus, would the results better favor modularity or interdependency? These are important queries regarding the nature of GRN architecture and their resolution may require extensive re-examination of “settled” results using newly available methods.

## 4. Challenges for the Future

While we have focused here on these two specific examples, other comparative studies of mechanisms of GRN adaptation and evolution also suggest that, as is the case with the *Drosophila* and *Heliconius* pigmentation GRNs, re-wiring of GRNs is a common cause of network co-option. Studies in plants, examining the diversity in leaf complexity in tomato plants and two related wild species [62]; in echinoderms, where GRNs for endomesoderm specification of both sea stars and sea urchins have been assembled (Figure 1; [7,63]); and in cichlids, where the visual system, an adaptive trait, has been studied in East African populations [64], all point to mutations in *cis*-regulatory sites as powerful drivers of evolutionary change [7,62,63,64]. These studies conjecture that variation and diversification rely on repeated changes at key nodes in GRN subcircuits, likely via modifications of specific CRMs. However, the evolutionary process is complex and, as demonstrated in the examples discussed above, varied mechanisms can lead to similar ends.

Focusing on just a specific subcircuit can mask this underlying mechanistic complexity [8]. Often, changes observed at the subcircuit level are the result of changes at a higher tier in the GRN. This leads to one of the biggest challenges in studying GRN evolution: teasing apart *cis*-regulatory changes from *trans*-regulatory changes. Protein-coding changes play a clear role in GRN evolution [65,66]. However, apparent changes in the *trans*-environment can be due to such “true” *trans*-specific changes, i.e., changes in the coding sequence of a transcription factor, or “pseudo” *trans* changes, where a *cis*-regulatory change modulates the transcription factor’s expression. From the perspective of a downstream subcircuit, both manifest as *trans*-effects: pseudo *trans* changes will resemble true *trans*-regulatory changes until examination of a higher tier in the GRN architecture reveals the responsible *cis*-regulatory change [6,67,68,69]. Hughes et al. [70] clearly establish the importance of a combination of *cis*- and *trans*-evolution in the diversification of the *Drosophila* pigmentation GRN, but do not clearly distinguish between the more likely scenario of extensive “pseudo” *trans* changes—changes to the *trans* environment due to *cis*-regulatory changes elsewhere in the GRN—and “true” *trans* changes at the TF level. Indeed, without knowledge of the full GRN, it is difficult to disambiguate these two alternatives, underscoring the importance of having a fully-defined GRN with known genes, CRMs, and transcription factor-DNA interactions.

The studies discussed above benefited from the ability to perform detailed sequence alignment of the relevant non-coding regions, allowing for homologous CRMs to be easily identified and enabling base pair-level differences in binding sites to be detected. This has provided tremendous insight into GRN evolution, especially at the level of terminal, non-regulatory subcircuits (“gene batteries”) [2,4]. Understanding evolutionary changes at higher, more regulatory GRN levels, however, may require comparisons between more extensively diverged species. Unfortunately, as evolutionary distance increases, sequence alignment and binding site conservation become increasingly challenging to parse out, making it difficult to identify homologous CRMs and relevant sequence changes. Important concerns going forward include determining the best methods to use to bridge the gap between closely related and more highly diverged species and identifying the right sets of species with the right degrees of divergence.

Promisingly, recent technical advances have made it easier to work with traditionally non-model species and their more highly diverged relatives. ATAC-seq has emerged as an affordable method for CRM detection starting from small cell populations [71,72], and computational CRM discovery approaches such as SCRMshaw have shown effectiveness in predicting putatively homologous CRMs in a cross-species manner [73,74,75]. As more species are sequenced, this increases the ability to find relevant CRMs for the GRNs being studied, although the quality of genome assembly and annotation and the need for robust gene orthology mapping remain important limitations. CRISPR/Cas9-mediated genome engineering, in particular, makes testing predicted CRMs and conducting gene perturbation studies significantly more feasible in a wide selection of species [76,77]. The increasing efficiency and availability of single-cell methods will make it easier to zero in on the transcription factors and CRMs that are active in GRN subcircuits in specific cell populations [78,79].

Through their modularity and their hierarchical structure, GRNs provide a framework for identifying evolutionary changes and their mechanisms. As we shift from looking within a single species to characterizing GRN differences between species, especially as interspecific distances increase, unraveling the tiers in the increasingly complex GRN architecture takes on an ever-greater importance. While specific to the *Drosophila* and *Heliconius* pigmentation GRNs, the examples discussed here illustrate common themes of network co-option and effector diversification, and they raise new questions about the nature of CRM modularity and redundancy. Above all, they highlight the importance of taking a broad-based yet comprehensive approach, encompassing both multiple species and multiple techniques, to reveal the *cis*- and *trans*-regulatory changes in GRNs that drive evolution.

## Data Availability

Data sharing not applicable, no new data generated.
